# IL-10/STAT3 is reduced in childhood obesity with hypertriglyceridemia and is related to triglyceride level in diet-induced obese rats

**DOI:** 10.1186/s12902-018-0265-z

**Published:** 2018-06-13

**Authors:** Yuesheng Liu, Dong Xu, Chunyan Yin, Sisi Wang, Min Wang, Yanfeng Xiao

**Affiliations:** 1 Department of Pediatrics, The Second Affiliated Hospital of Xi’an Jiaotong University, 157 Xiwu Road, Xi’an, Shaanxi, 710061 People’s Republic of China; 2 Tongji Hospital, Tongji Medical College, Huazhong University of Science and Technology, Wuhan, Hubei 430030 People’s Republic of China

**Keywords:** Childhood obesity, High-fat diet, IL-10, JAK-STAT, Triglyceride

## Abstract

**Background:**

The prevalence of childhood obesity and obesity-related metabolic disorder such as dyslipidemia has sharply increased in the past few decades. Chronic low-grade inflammation is associated with the development of comorbidities and poor prognosis in obesity. This study aims to evaluate interleukin-10 (IL-10) in childhood obesity with hypertriglyceridemia.

**Method:**

We evaluated IL-10 and signal transducer and activator of transcription 3 (STAT3) mRNA expression in adipose tissue (AT) as well as serum IL-10 in 62 children of 3 groups and in high-fat diet (HFD) induced obese rat. Expression of IL-10 and STAT3 protein in AT of diet-induced obese rats were examined over feed period.

**Results:**

Adipose IL-10 and STAT3 mRNA expression and serum IL-10 reduced in obese children with hypertriglyceridemia and in HFD obese rats. The protein expression of IL-10 and STAT3 decreased in AT of obese rats compared with the control rats at end time. Expression of IL-10 mRNA was negatively correlated to TG and LDL-C levels, and positively correlated to HDL-C, adiponectin and serum IL-10 levels.

**Conclusions:**

IL-10 expression and its downstream JAK-STAT pathway are down-regulated in obese children with hypertriglyceridemia and in HFD obese rats.

**Electronic supplementary material:**

The online version of this article (10.1186/s12902-018-0265-z) contains supplementary material, which is available to authorized users.

## Background

In the past few decades, with the rapid economic growth and shifts in diet and lifestyle, the prevalence of overweight and obesity has also sharply increased in children and adolescents in both developed and developing countries [1–3]. In 2010, the overweight and obesity combined prevalence had reached 19.2% among Chinese children and adolescents aged 7–18 years [4]. As the prevalence of obesity and associated disease continues to rise, social costs also escalate rapidly [5]. Childhood obesity is strongly associated with several metabolic abnormalities such as dyslipidemia, hypertension, impaired fasting glucose, and metabolic syndrome (MetS) [6–8]. Low serum high-density lipoprotein cholesterol (HDL-C) levels, increased low-density lipoprotein cholesterol (LDL-C), high triglycerides (TG) levels are frequent metabolic disorders in childhood obesity and hypertriglyceridemia is the most frequent [9]. These health problems not only affect metabolism and psychosocial conditions in the short term, but also lead to an increased risk of cardiovascular disease in adulthood. Therefore, it is important to determine effective measures for the prevention of obesity and obesity-related hypertriglyceridemia in children.

Obesity may be accompanied by chronic low-grade inflammation with unclear triggers [10]. Alterations of adipokines secreted by adipose tissue (AT) and several further cytokines are thought to contribute to a low-grade inflammation [11]. Some studies have indicated that the pro-inflammatory cytokines, such as IL-6, could promote lipolysis [12, 13]. Besides, a lot of adipose tissue-related anti-inflammatory factors have been suggested to be involved in the pathogenesis of systemic inflammation in obesity. IL-10 is known as one of the most important anti-inflammatory and immunosuppressive cytokines that mainly acts on monocytes. The main source of IL-10 in vivo appears to be immune cells, such as monocytes, macrophages, and different T-cell subsets [14]. Previous study has shown that monocyte subsets display lower IL-10 expression during childhood obesity [15]. STAT3 is one of the downstream pathway gene of IL-10. JAK-STAT3 pathway is associated with on the development of obesity-associated disorders such as leptin resistance and insulin resistance [16]. However, the regulation and role of IL-10 and JAK-STAT3 pathway in adipose tissue are not completely understood in childhood obesity. Are these factors alterant in childhood obesity and obesity-related disorder?

In the present study, we observed that childhood obesity with hypertriglyceridemia were accompanied by a predominant inflammatory state with a decrease of IL-10 expression in SAT. In addition, we conducted a diet induced obese rat study to validate it. Our hypothesis is that decrease of IL-10 in AT is associated with triglyceride metabolism, which could contribute to the pathogenesis of childhood obesity. Clarifying the role of IL-10 in lipid metabolism may provide a new therapeutic strategies in obesity-association disorders.

## Methods

### Study population

Sixty-two children undergoing surgery for nonmalignant and non-inflammatory diagnose were recruited. According to the BMI reference norm for Chinese children and adolescences, subjects were considered to be obese when the BMI exceeded 95th percentile of the norm [17]. BMI standard deviation score (SDS-BMI) were calculated according to WHO BMI-for-age (5–19 years) [18].Subjects were diagnosed with hypertriglyceridemia (serum TG level > 1.7 mmol/L) according to the new International Diabetes Federation definition for Chinese people [19]. The study protocol was approved by the Ethical Committee of Xi’an Jiaotong University. Written informed consent was obtained from all guardians of children participating in the study. The study was conducted according to the Declaration of Helsinki.

### Adipose tissue and blood sample

Immediately after skin incision and subcutaneous tissue exposure during the surgery, AT were separated bluntly, washed in cold PBS, and stored at liquid nitrogen for quantitative real-time PCR (qRT-PCR). Fasting venous blood sample from each participant was collected and stored at − 80 °C for biochemical measurements.

### Animals

One hundred forty-four male clean Sprague-Dawley rats from the age of 3 weeks after weaned were purchased in Animal Experimental Center of Xi’an Jiaotong University. Rats were housed under a 12:12 h light-dark cycle with well ventilation and constant temperature (26 ± 1 °C). Animals were adapted to laboratory lighting and feeding condition for 1 week before experiment. Rats were allowed free access to food and drinking water. After adaptation, rats were randomly divided into two groups. The control group (*n* = 48) was given normal diet (ND). The other group (*n* = 96) were fed with high-fat diet (HFD). The HFD was prepared by mixing 2 g cholesterol and 15 g lard oil with 83 g ND. The HFD consists of 44.9% fat, 35.1% carbohydrate as starch, 20.0% protein as casein as the energy sources while the ND contained 20.8% fat, 60.9% carbohydrate, 18.3% protein. The body weight and food intake of each rat were measured once and twice per week. Rats with weight 20% higher than average weight of control group were regarded of obese rats. Obesity evaluation was conducted in 8th, 16th, 20th and 24th week, respectively. 12 ND rats and 12 HFD obese rats were sacrificed under anesthesia by an intraperitoneal injection of chloral hydrate in 8th, 16th, 20th week, respectively. In 24th week, all of the remaining rats (*n* = 16) were sacrificed in the same way, while non-obesity HFD rats were excluded. All animal experiment protocols were approved by the Ethics Committee of the Xi’an Jiaotong University and were performed according to the Institutional Animal Care and Use Committee of Xi’an Jiaotong University. All experimental procedures conformed to the European Guidelines for the care and use of Laboratory Animals (directive 2010/63/EU).

### Biochemical measurements

Fasting blood glucose (FBG), total cholesterol (TC), triglycerides (TG), high-density lipoprotein cholesterol (HDL-C) and low-density lipoprotein cholesterol (LDL-C) were measured enzymatically using an autoanalyzer (Hitachi 747). Plasma free fatty acids (FFA) concentrations were determined using a commercial enzymatic kit (Applygen). Fasting Insulin was assayed by RIA (BeiFang systems). Plasma levels of leptin and adiponectin were also measured by Elisa kits (Excell). Serum IL-10 concentrations were measured using Elisa kits (Wuhan Boster Biotechnology Inc.).

### Immunohistochemistry

After rats were sacrificed, perirenal and epididymal AT were quickly removed, weighed, frozen immediately in liquid nitrogen, and kept at − 80 °C for the analysis of mRNA expression or fixed with 4% paraformaldehyde for histological examination. AT sections (5 μm) were stained with IL-10 and P-STAT3 (Immunoway) respectively. The protein–antibody immune complexes were detected with horseradish peroxidase-conjugated secondary antibodies and enhanced chemiluminescence reagents (Millipore). The average optical density (AOD) of each protein in sections were detected by Image-Pro plus 6.0.

### RNA isolation and quantitative real-time PCR

Total RNA was isolated using Trizol reagent (Invitrogen). Then cDNA was synthesized using a high-capacity Reverse Transcription kit (Takara) and diluted in DNase-free water before use. QRT-PCR was performed on an ABI HT7500 PCR machine using the comparative Ct-method with SYBR Green PCR kit (Takara). Relative mRNA expression levels of target genes were normalized to GAPDH. The following primers were used: IL-10 (human), 5’-TGTCATCGATTTCTTCCCTGT-3′ and 5’-GGCTTTGTAGATGCCTTTCTCT-3′; IL-10 (rat), 5’-CAGACCCACATGCTCCGAGA-3′ and 5’-CAAGGCTTGGCAACCCAAGTA-3′; STAT3 (human), 5’-TTTGAGACAGAGGTGTACCACCAAG-3′ and 5’-ACCACAGGATTGATGCCCAAG-3′; STAT3 (rat) 5’-TGTGACACCAACGACCTGC-3′ and 5’-TCCATGTCAAACGTGAGCGA-3′; GAPDH (human), 5′ -AATGGACAACTGGTCGTGGAC-3′ and 5′ -CCCTCCAGGGGATCTGTTTG-3′; GAPDH (rat), 5′ -GGTGGACCTCATGGCCTACA-3′ and 5′ -CTCTCTTGCTCTCAGTATCCTTGCT-3′.

### Statistical analysis

The data was expressed as the mean ± standard error. Differences between the two groups were analyzed with a Student’s t-test. Differences between the serum lipids at the different feed periods were assessed using a two-way ANOVA. Pearson’s correlations between IL-10 and other parameters were calculated. The differences were considered statistically significant at *p* < 0.05. All analyses were performed by SPSS 22.0 (SPSS Inc.).

## Results

### Inflammation and metabolic disorders associated with childhood obesity

The clinical features of non-obese controls (*n* = 31), obese children without hypertriglyceridemia (*n* = 17) and obese children with hypertriglyceridemia (*n* = 14) are shown in Table 1. Anthropometric evaluation showed that BMI, SDS-BMI, waist circumference, waist-hip Ratio and SBP of obese children with/without hypertriglyceridemia were higher than non-obese children. For biochemical measurements, obese children with/without hypertriglyceridemia also showed an increase in fasting insulin, HOMA-IR, TG, FFA and LDL-C, and concomitant reduction in HDL-C levels.

**Table 1:** Clinical features of study groups

	Non-obese (*n* = 31)	Obese without hypertriglyceridemia (*n* = 17)	Obese with hypertriglyceridemia (*n* = 14)
Sex (male/female)	21/10	11/6	9/5
Age (years)	8.14 ± 1.69	8.72 ± 2.36	8.59 ± 1.78
BMI (kg/m^2^)	15.13 ± 1.19	22.19 ± 2.77*	22.85 ± 2.19*
SDS-BMI	−0.52 (−1.41, 0.38)	2.59 (2.02, 3.08)*	2.66 (1.99,3.39)*
Waist circumference (cm)	55.48 ± 3.84	71.52 ± 11.59*	71.65 ± 9.3*
Waist-hip Ratio	0.88 ± 0.06	0.92 ± 0.05*	0.92 ± 0.05*
SBP (mmHg)	95 ± 7	100 ± 7*	103 ± 9*
DBP (mmHg)	59 ± 6	62 ± 5	61 ± 10
FBG (mmol/L)	4.67 ± 0.40	4.72 ± 0.42	4.76 ± 0.63
Fasting insulin (μU/mL)	5.04 ± 3.34	12.96 ± 4.36*	13.03 ± 5.07*
HOMA-IR	0.96 ± 0.41	2.59 ± 1.21*	2.67 ± 1.10*
TC (mmol/L)	3.43 ± 0.66	3.80 ± 0.36	3.71 ± 0.79
TG (mmol/L)	0.86 ± 0.40	0.87 ± 0.32*	2.79 ± 0.95*^,^**
FFA (μmol/L)	367.22 ± 50.79	416.62 ± 44.72*	445.36 ± 60.38*
HDL-C (mmol/L)	1.37 ± 0.19	1.15 ± 0.11*	1.04 ± 0.14*
LDL-C (mmol/L)	1.67 ± 0.48	2.11 ± 0.47	2.33 ± 0.13*^,^**
Leptin (ng/mL)	10.56 ± 8.99	30.80 ± 15.11*	26.02 ± 10.89*
Adiponectin (μg/mL)	3.35 ± 1.20	2.73 ± 1.33*	2.57 ± 1.09*^,^**
leptin-to-adiponectin ratio (ng/μg)	3.45 ± 2.11	11.56 ± 5.90*	14.71 ± 9.18*^,^**

*BMI* body mass index, *SDS-BMI* standard deviation scores BMI, *SBP* systolic blood pressure, *DBP* diastolic blood pressure, *FBG* fasting blood glucose, *TC* total cholesterol, *TG* triglycerides, *FFA* free fatty acids, *HDL-C* high-density lipoprotein cholesterol, *LDL-C* low-density lipoprotein cholesterol. *p < 0.05 vs. Non-obese. **p < 0.05 vs. Obese without hypertriglyceridemia

Adiponectin and leptin were measured to characterize inflammation status. Obese children with/without hypertriglyceridemia showed an increase of leptin and leptin-to-adiponectin ratio, and a decrease in adiponectin (Table 1). Moreover, we observed that obese children with hypertriglyceridemia showed a decrease in adiponectin compared with children without hypertriglyceridemia (Table 1).

### IL-10 decreased in adipose tissue and serum of obese children with hypertriglyceridemia

**Fig. 1:**
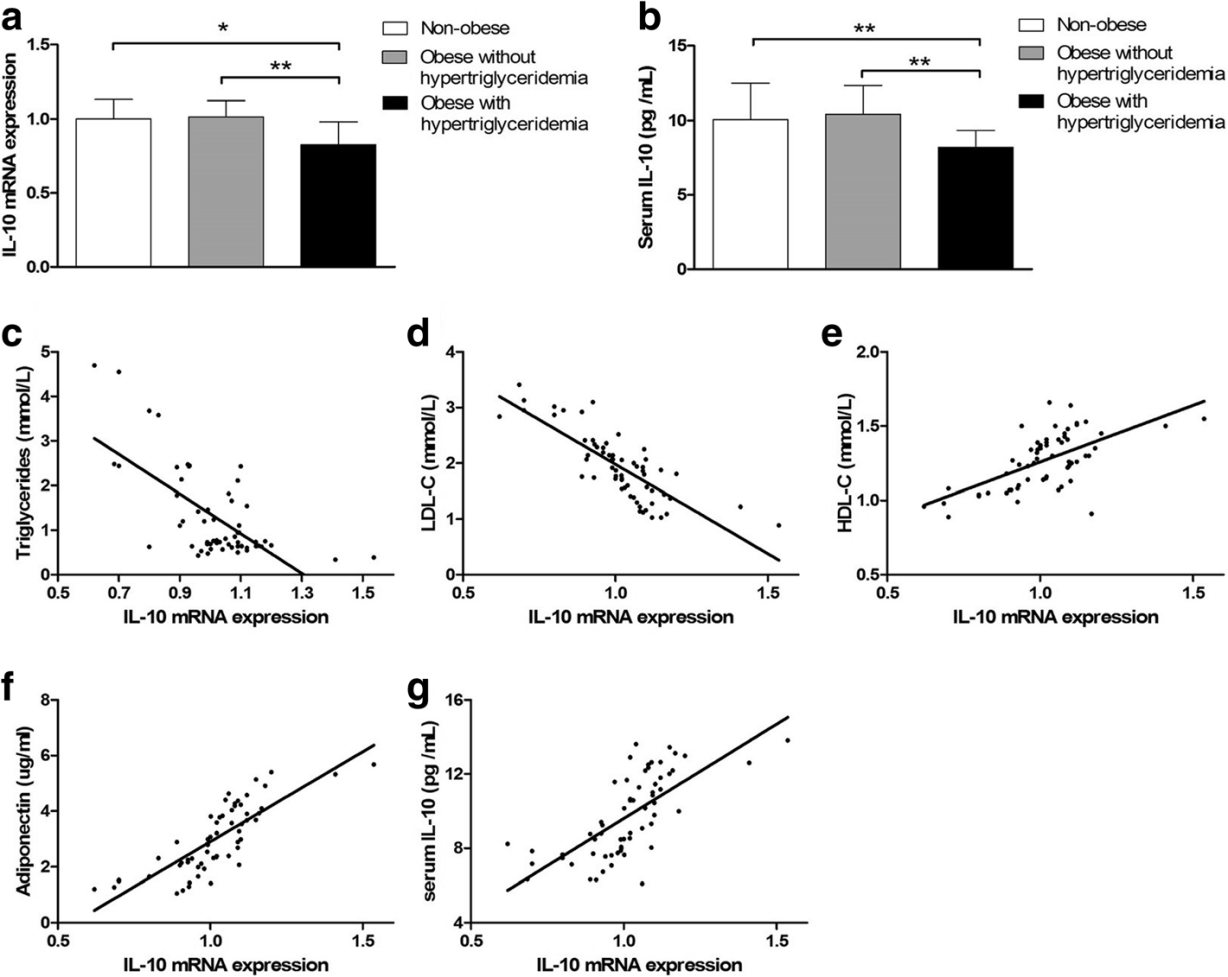
Evaluation of mRNA expression in adipose tissue and serum levels of IL-10. **a** Adipose IL-10 mRNA expression and (**b**) serum IL-10 decreased in obese children with hypertriglyceridemia (*n* = 14). *P < 0.01 vs. non-obese controls (*n* = 31). **P < 0.01 vs. obese without hypertriglyceridemia (*n* = 17). IL-10 mRNA expression was negatively correlated to (**c**) TG and (**d**) LDL-C levels, and positively correlated to (**e**) HDL-C, (**f**) Adiponectin and (**g**) serum IL-10 levels. The data are presented as mean ± SD

QRT-PCR and Elisa showed that IL-10 mRNA in AT and serum IL-10 levels were similar between non-obese and obese group (Additional file 1: Table S1). However, when compared with the non-obese and the obese without hypertriglyceridemia group, IL-10 mRNA expression in AT and serum IL-10 levels were lower in obese children with hypertriglyceridemia (Fig. 1a, b). Furthermore, the expression of IL-10 mRNA in AT was negatively correlated to TG and LDL-C levels, and positively correlated to HDL-C, adiponectin and serum IL-10 levels in this cohort (Fig. 1c-g).

### Serum lipids is increased in HFD obese rats

**Fig. 2:**
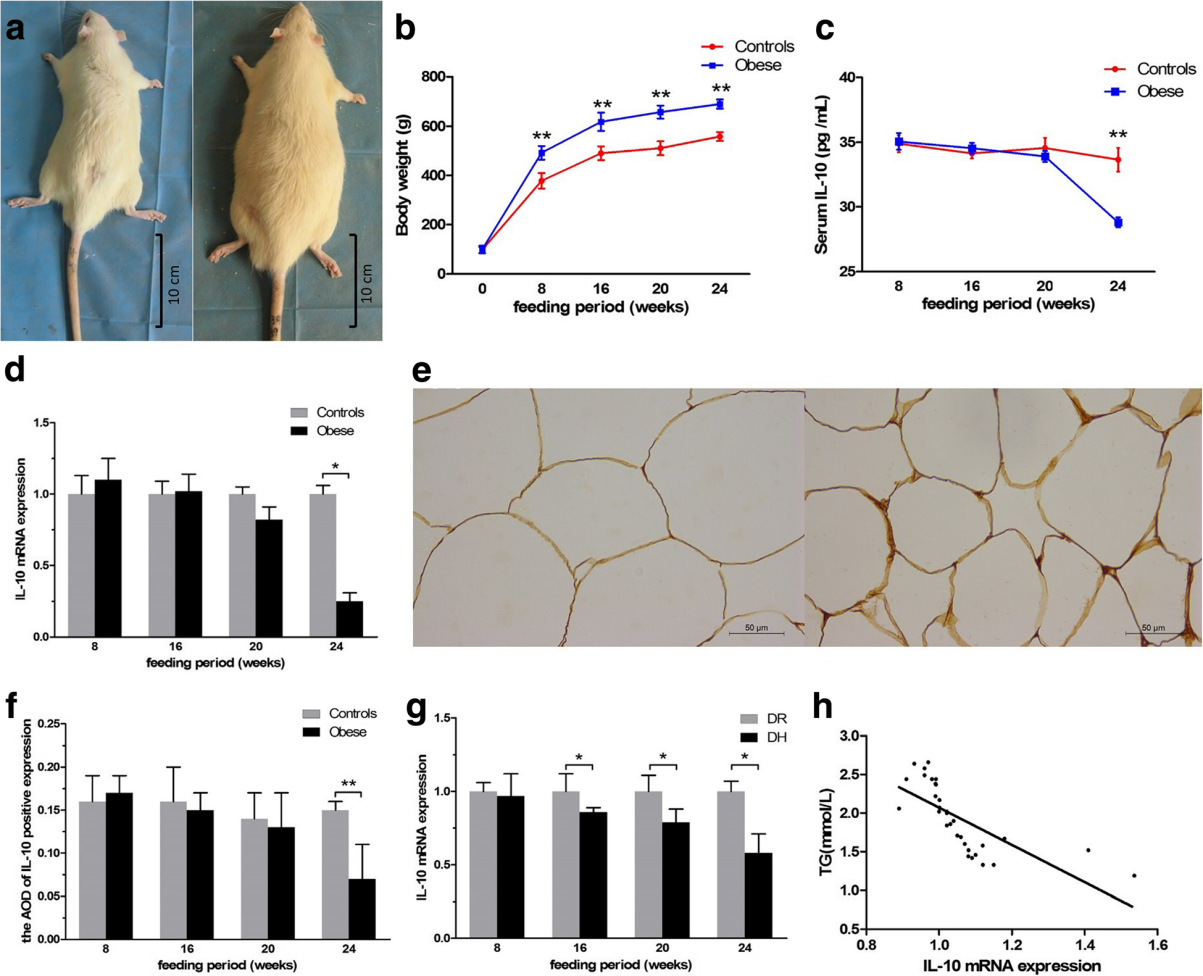
Evaluation of IL-10 in HFD obese rats. **a** Photographs of representative rats of ND group and HFD group after 16 weeks. HFD Obese rat (right) was larger in size with yellow pelage compared with ND rat (left). **b** Mean body weight in each group over a period of 24 weeks. **c** The serum IL-10 concentrations was reduced in HFD obese rats in 24th week. **d** IL-10 mRNA expression of adipose tissue was reduced in HFD obese rats in 24th week. **e** Histological sections were stained with antibody against IL-10 in ND controls group (left) and HFD obese group (right) in 24th week. **f** The AOD of IL-10 protein in adipose tissue of HFD obese rats was lower in 24th week. **g** After 16th week, IL-10 mRNA expression of DH group was lower than DR group. **h** IL-10 mRNA in adipose tissue was negatively correlated to serum TG levels. *P < 0.05, **P < 0.01. The data are presented as mean ± SD

Physical appearance and body weights of rats were measured to confirm the HFD-induced obese phenotype. Rats fed with HFD with the end body weight 20% higher than that in the ND group were considered as obese rats (obesity rate was 86.2%). Obese rats were significantly larger in size, with yellow pelage (Fig. 2a). Rats fed with HFD showed a significant increase in mean body weight as compared to the ND rats after 8th week (Fig. 2b). After 20th week, serum FFA and TG in HFD obese rats were significantly higher than those in ND rats, remarkably in 24th week. Serum TC level was significantly higher compared with ND rats in 24th week (Table 2). Serum lipids is increased in HFD obese rats in 24th week.

**Table 2:** Serum lipids of ND and HFD rats

week	TG(mmol/L)		TC(mmol/L)		FFA(μmol/L)	
	ND	HFD	ND	HFD	ND	HFD
8	1.64 ± 0.23	1.77 ± 0.44	1.29 ± 0.26	1.27 ± 0.53	305.23 ± 8.35	307.43 ± 11.87
16	1.51 ± 0.31	1.79 ± 0.31	1.25 ± 0.12	1.30 ± 0.44	310.56 ± 7.42	315.95 ± 4.20
20	1.76 ± 0.34	2.06 ± 0.11^a^	1.33 ± 0.42	1.37 ± 0.59	308.73 ± 11.64	321.89 ± 6.12^a^
24	1.61 ± 0.29	2.67 ± 0.94^a,b^	1.36 ± 0.31	1.76 ± 0.53^a,b^	302.64 ± 10.14	329.32 ± 4.15^a,b^

*TG* triglyceride, *TC* total cholesterol, *FFA* free fatty acids. ^a^ *p* < 0.05 vs. ND. ^b^ *p* < 0.05 vs. 8th week. 8th, 16th, 20th week *n* = 12, 24th week n = 16

### IL-10 is reduced in HFD obese rats

In HFD obese rats, the serum IL-10 concentrations was reduced in 24th week (Fig. 2c). When compared with controls, IL-10 mRNA expression in AT was lower in HFD obese rats in 24th week (Fig. 2d). Immunohistochemical staining showed that IL-10 protein expression was reduced in HFD obese rats in 24th week (Fig. 2e). Moreover, the AOD of IL-10 protein expression in AT of HFD rats was significantly reduced compared with ND rats (Fig. 2f). QRT-PCR and immunohistochemical staining showed that the expression of IL-10 in AT was reduced in HFD obese rats. According to the sorting of TG increments, HFD rats at the top 1/3 (*n* = 4) of TG were considered as diet-induce hypertriglyceridemia (DH) group and HFD rats at the lower 1/3 (n = 4) of TG were considered as diet-induce hypertriglyceridemia resistance (DR) group. After 16th week, IL-10 mRNA expression of DH rats was lower than DR rats. Additionally, IL-10 mRNA in AT was negatively correlated to serum TG levels (Fig. 2h).

### STAT3 is reduced in obese children with hypertriglyceridemia and HFD obese rats

**Fig. 3:**
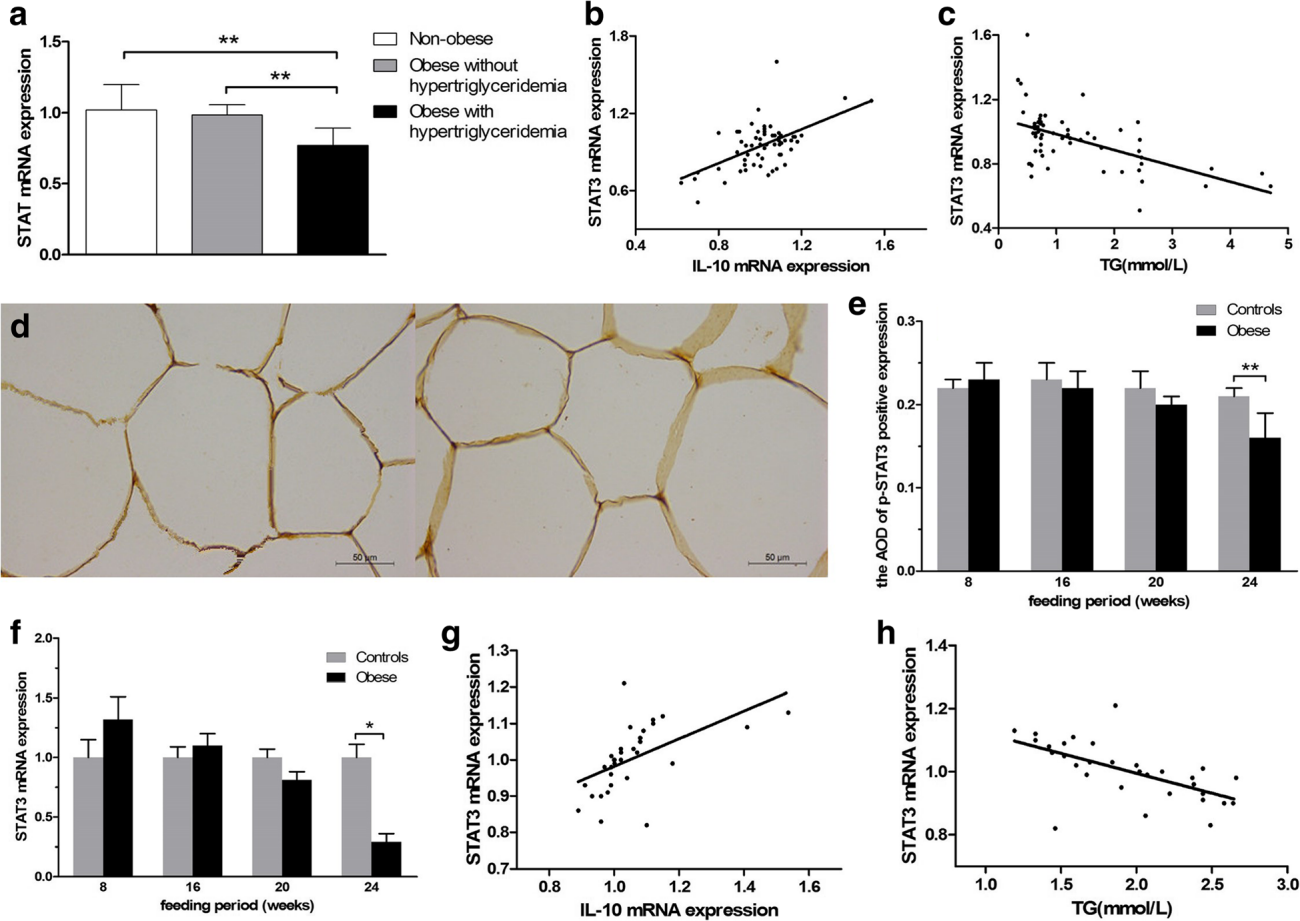
Evaluation of STAT3 expression. **a** STAT3 mRNA expression were reduced in obese children with hypertriglyceridemia. **b** Adipose STAT3 mRNA expression was positively correlated to IL-10 expression in adipose tissue of children. **c** Adipose STAT3 mRNA expression was negatively correlated to serum TG levels in children. **d** Histological sections were stained with antibody against P-STAT3 in ND controls group (left) and HFD obese group (right) in 24th week. **e** The AOD of P-STAT3 protein in adipose tissue of HFD obese rats was lower in 24th week. **f** STAT3 mRNA expression of adipose tissue was lower in HFD obese rats in 24th week. **g** Adipose STAT3 mRNA expression was positively correlated to IL-10 expression in rats. **h** Adipose STAT3 mRNA expression was negatively correlated to serum TG levels in rats. *P < 0.05, **P < 0.01. The data are presented as mean ± SD

QRT-PCR showed that when compared with the non-obese and the obese without hypertriglyceridemia group, STAT3 mRNA expression were lower in obese children with hypertriglyceridemia (Fig. 3a). Immunohistochemical staining showed that P-STAT3 protein expression was reduced in HFD obese rats when compared with ND rats (Fig. 3d, e). QRT-PCR showed the similar results that STAT3 mRNA expression of AT was reduced in HFD obese rats in 24th week when compared with ND rats (Fig. 3f). In addition, STAT3 mRNA expression in AT was positively correlated to IL-10 expression, and negatively correlated to TG levels both in children and rats (Fig. 3b, c, g, h).

## Discussion

Obesity is a chronic and persistent low inflammatory condition triggered by the immune response and by the metabolic regulator molecules. The mechanisms behind this association are still unclear. However, it is possible that the key inflammatory regulators also play important role in regulating inflammatory responses. IL-10 is anti-inflammatory cytokine and contributed with low inflammatory condition in childhood obesity [20, 21]. However, the effect of IL-10 on pathogenesis of childhood obesity and related disorder is unclear.

In the present study, we evaluated the adipose and circulating IL-10 levels and their association with the childhood obesity and obesity–related hypertriglyceridemia in both a cross-sectional study of humans and in a rat study. Clinical and laboratorial characteristics of obese children with or without hypertriglyceridemia and controls showed that obese children with hypertriglyceridemia have lower adiponectin and higher leptin-to-adiponectin ratio when compared to the controls and children without hypertriglyceridemia. These results are consistent with the adult cohort that adiponectin level is lower in hypertriglyceridemia and independent relation to hypertriglyceridemia [22] which indicated higher grade of inflammatory status and adipose tissue physiology. Previous study reported that in obese women the prevalence of the metabolic syndrome was associated with low circulating IL-10 levels [23]. Moreover, IL-10 expression was not reduced in obesity with normal blood triglycerides adult cohort [24], which is similar to our observation. However, we innovatively demonstrated that obese children with hypertriglyceridemia showed lower adipose and serum IL-10 compared to obese children without hypertriglyceridemia and non-obese children. Thus, IL-10 in adipose tissue and circulation is down-regulated in obese children with hypertriglyceridemia.

Previous study reported that increased IL-10 in patients with lymphoproliferative disorder causes elevated triglycerides, low LDL-C and HDL-C deficiency, and IL-10 is thus a potent modulator of lipoprotein levels [25]. HDL-C decreased significantly in IL-10-deficient mice compared to WT mice [26], consistent with effects of systemic inflammation in decreasing HDL-C [27]. We evidenced that mRNA expression of IL-10 in SAT of children was negatively correlated to TG and LDL-C levels, and positively correlated to HDL-C. Coincide with the human study, the negative correlation between adipose IL-10 and serum TG was indicated in rats study. Lipid abnormalities occur in autoimmune and inflammatory disorders with incompletely understood mechanism, but the inflammatory cytokines have been implicated to play an important role [27]. Several cytokines that induce lipolysis, including TNF, IFN-α, and IFN-γ, produce a marked decrease in hormone-sensitive lipase (HSL) mRNA [28]. IL-1 effect on serum TG levels is attributable to enhanced hepatic FA synthesis and TG secretion [29]. Thus, dysregulation of IL-10 is directly or indirectly associated with pathogenesis of obesity-related hypertriglyceridemia.

It is appropriate to use high energy and high fat diet to induce obesity model in obese rats, which have been widely used to study the pathogenesis and progression of obesity because they have the similar gene expression profiling with obese humans [30, 31]. To observe the adipose and circulating IL-10 levels and their association with obesity, we established a high-fat diet induced obesity rat model with aggravated lipids disorders, of which serum TG, FFA and TC were increased. Consistent with previous study, we observed showed that serum IL-10 was reduced in obese rats induced by HFD [32]. HFD-induced obesity is accompanied by inflammatory status of IL-10 down-regulation in tissue. In heart tissue, IL-10 was found to be down-regulated in HFD rats and up-regulated in HFD-exercise groups [33]. We focused on adipose tissue and evidenced that the levels of IL-10 decreased in adipose tissue of obese rats after a long HFD feed period (in 24th week). Interestingly, after dividing the obese rats into subgroup according to the TG increments ranking, we observed that IL-10 mRNA expression of diet-induce hypertriglyceridemia rats was lower than hypertriglyceridemia resistance rats in the earlier feed period (in 16th week) along with weight gain in HFD rats. Adipose tissue dysfunction with chronic inflammation is considerably associated with the development of insulin resistance, type 2 diabetes, and cardiovascular diseases [34]. Dysfunction of adipocytes, changes in metabolic profile or immune cells profile have been indicated in obesity [35, 36], which may result in the alteration of IL-10 expression and chronic inflammation in AT.

Recent studies have demonstrated that IL-10 can exert anti-inflammatory effects through the JAK-STAT3 pathway: After IL-10 binding to the receptor on the target cell membrane, the tyrosine kinase 2 (Tyk2), which is a subtype of the JAK protein family coupled with the IL-10 receptor, is further activated; Stimulation of Tyk2 with IL-10 leads to activation of STAT3 by its SH2 domain tyrosine phosphorylation [14, 37, 38]. One of the primary determinants of plasma TG is lipoprotein and the various proteins, such as apoC-II, apoC-III, ANGTPL3, that regulate lipoprotein. [39–41] Overexpression of apolipoprotein A-I significantly increased the phosphorylation of STAT3 as well as its upstream JAK2 kinase, which increased serum TG, TC and HDL-C levels [42]. It is reported that interleukin-10 gene transfer inhibits atherogenesis in ApoE-deficient mice through a STAT3-dependent anti-inflammatory pathway [43]. Present study has shown that adipose STAT3 expression was decreased in obese children with hypertriglyceridemia and in HFD obese rats. Moreover, STAT3 mRNA expression in AT was positively correlated to IL-10 expression, and negatively correlated to TG levels. We therefore concluded that IL-10/JAK-STAT pathway is associated with obesity-related hypertriglyceridemia, and the pathogenesis mechanism require further study.

We recognize that our research has some limitations. In the present study, the clinical sample size for evaluation of IL-10 expression was still small and a larger sample cohort is needed for verification. In addition, we observed and hypothesized that IL-10 participated in triglyceride metabolism associated with JAK-STAT pathway in HFD obese rats. However, the molecular regulation mechanisms of these potential regulators in childhood obesity still require to be investigated in vivo or vitro.

## Conclusion

We highlight the decrease of IL-10 expression and its downstream JAK-STAT pathway in obese children with hypertriglyceridemia and in HFD obese rats, which indicated that IL-10 might have a protective effect on the lipid metabolic disorders, particularly hypertriglyceridemia. We believe that these alternations exacerbate the inflammatory process and dysfunction in adipose tissue, contributing to dyslipidemia. Thenceforward, the observation of IL-10 as key cytokine in obesity-related metabolic disorders opens perspectives for designing new therapeutic strategies in to treat childhood obesity and its consequences.

## Additional file

Additional file 1: **Table S1.** mRNA expression in adipose tissue and serum levels of IL-10. (DOCX 17 kb)

## Abbreviations

AOD: Average optical density.
CHD: Coronary heart disease
DBP: Diastolic blood pressure
FBG: Fasting blood glucose
FFA: Free fatty acids
HDL-C: High-density lipoprotein cholesterol
HFD: High-fat diet
HSL: Hormone sensitive lipase
JAK: Janus Kinase
LDL-C: Low-density lipoprotein cholesterol
ND: Normal diet
SAT: Subcutaneous adipose tissue
SBP: Systolic blood pressure
STAT: Signal transducers and activators of transcription
TC: Total cholesterol
TG: Triglyceride
TG: Triglycerides
