# Supplementary material for: IL-10/STAT3 is reduced in childhood obesity with hypertriglyceridemia and is related to triglyceride level in diet-induced obese rats
Source: BMC Endocr Disord. 2018 Jun 13;18:39. doi: 10.1186/s12902-018-0265-z (PMC5998569; doi:10.1186/s12902-018-0265-z)
Supplement: Supplementary file 1 — Table S1. mRNA expression in adipose tissue and serum levels of IL-10. (DOCX 17 kb) [file 12902_2018_265_MOESM1_ESM.docx]

**IL-10/STAT3 is reduced in childhood obesity with hypertriglyceridemia and is related to triglyceride level in diet-induced obese rats**

Yuesheng Liu^1^, Dong Xu^2^, Chunyan Yin^1^, Sisi Wang^1^, Min Wang^1^ and Yanfeng Xiao^1,*^

^1^ The Second Affiliated Hospital of Xi’an Jiaotong University, Xi’an, Shaanxi, People’s Republic of China.

^2^Tongji Hospital, Tongji Medical College, Huazhong University of Science and Technology, Wuhan, Hubei, People’s Republic of China.

^*^Corresponding author (Email: [xiaoyanfeng0639@sina.com](mailto:xiaoyanfeng0639@sina.com))

Table S1 mRNA expression in adipose tissue and serum levels of IL-10

|  | Non-obese (n = 31) | Obese (n=31) | *P* value |
| --- | --- | --- | --- |
| IL-10 mRNA relative expression | 1 ± 0.13 | 0.93 ± 0.16 | 0.053 |
| STAT mRNA relative expression | 1 ± 0.21 | 1.06 ± 0.10 | 0.136 |
| serum IL-10 levels | 10.07 ± 2.42 | 9.42 ± 1.95 | 0.249 |
